# Potential of *Lecanicillium uredinophilum* as a Biocontrol Agent of *Hemileia vastatrix*: A Review Compared with Other Biological Control Agents

**DOI:** 10.3390/biology15070589

**Published:** 2026-04-07

**Authors:** Jose Luis Pinedo-Mas, Eyner Huaman, Amilcar Valle-Lopez, Jamil Delgado Rafael, Raúl Vargas, Robin Oblitas-Delgado, Jhon Edler Lopez-Merino, Manuel Oliva-Cruz

**Affiliations:** Instituto de Investigación para el Desarrollo Sustentable de Ceja de Selva (INDES-CES), Universidad Nacional Toribio Rodríguez de Mendoza (UNTRM), Chachapoyas 01001, Peru; 7425007181@untrm.edu.pe (J.L.P.-M.); eyner.huaman@untrm.edu.pe (E.H.); vallelopezamilcar2002@gmail.com (A.V.-L.); 7629090521@untrm.edu.pe (J.D.R.); raul.vargas.epg@untrm.edu.pe (R.V.); robin.oblitas.epg@untrm.edu.pe (R.O.-D.); jhon03.lopez01@gmail.com (J.E.L.-M.)

**Keywords:** coffee leaf rust, mycoparasitism, fungal antagonists

## Abstract

Coffee leaf rust (*Hemileia vastatrix*) is a major constraint on coffee production, while reliance on chemical fungicides raises environmental concerns and may become less sustainable over time. This review critically analyzes the available evidence on the potential of *Lecanicillium uredinophilum* as a biological control agent against *H. vastatrix*, with comparative consideration of other microbial agents evaluated for coffee leaf rust management. A structured literature review was conducted using searches in Scopus and PubMed, complemented by manual searches and reference screening. The available evidence indicates that *L. uredinophilum* shows affinity for urediniospore structures and exhibits mycoparasitic activity against rust fungi under controlled conditions. However, direct evidence against *H. vastatrix* remains limited and is still concentrated mainly in laboratory and greenhouse studies. In comparison with more established biocontrol agents, *L. uredinophilum* should be regarded as a promising but still early-stage candidate whose practical relevance has not yet been validated. Future progress will depend on robust field-based studies, improved understanding of its mechanisms of action, evaluation of its environmental stability, and the development of viable formulations compatible with integrated disease management strategies.

## Simple Summary

Coffee leaf rust is one of the most damaging diseases affecting coffee production worldwide. It is commonly managed with chemical fungicides, but these products may create environmental problems and can become less effective over time. This review examines whether *Lecanicillium uredinophilum*, a fungus naturally associated with rust fungi, could be used as a safer biological alternative to help manage coffee leaf rust. The available studies show that this fungus can attack rust spores and other fungal structures under controlled conditions, which suggests that it has real potential as a biological control agent. However, most of the current evidence comes from laboratory and greenhouse studies, and there is still little proof that it works consistently under field conditions. Compared with other biocontrol agents already studied for coffee leaf rust, *L. uredinophilum* appears promising but remains at an early stage of development. More research is needed to confirm its effectiveness in coffee plantations, understand how it works in greater detail, and develop stable formulations that can be used by farmers. This information may support more sustainable coffee disease management in the future.

## 1. Introduction

Coffee is cultivated on approximately 10 million hectares distributed across about 12.5 million agricultural holdings worldwide, making it one of the most relevant crops in economic and social terms [1]. The coffee production chain provides direct and indirect livelihoods for more than 100 million people globally [2,3].

In recent years, the international coffee market has experienced sustained growth driven by its global productive and commercial expansion, with Brazil remaining the world’s leading producer [4]. Global coffee consumption is projected to increase at an annual rate of 1–2% in the coming years, while consumption in Latin America has risen significantly over the past decade [5]. In parallel, coffee has been consolidated as a product of high social and cultural value through global promotional strategies that reinforce consumer perceptions of quality, diversity, and production systems [6].

Nevertheless, the sustainability of coffee production is seriously threatened by the incidence of pests and diseases. At the global level, plant pathogens can reduce agricultural yields by 20–40%, depending on crop, region, and year [7]. In the particular case of coffee, rust caused by *H. vastatrix* represents one of the most destructive phytopathogens, capable of causing yield reductions that can reach ~35% in widespread epidemics, and may exceed 70% under severe local scenarios associated with heavy defoliation and compounding (polyetic) effects [8,9,10]. Beyond its productive impact, this disease significantly affects coffee cup quality, compromising the commercial value of the final product [11]. For example, in Tanzania, it has been estimated that nearly 50% of production costs can be directly associated with chemical control strategies targeting the two main fungal diseases of the crop, coffee leaf rust (CLR) and coffee berry disease [12].

Conventional management of CLR is primarily based on the use of preventive copper-based fungicides and systemic fungicides from the azole group, which have proven effective in controlling the disease [3]. However, their intensive and prolonged application can generate adverse effects, such as copper accumulation in soils and phytotoxicity in plants [13]. Additionally, many agrochemicals exhibit low biodegradability, leading to the persistence of toxic residues in soils and associated risks to human, animal, and soil ecosystem health [14]. Moreover, repeated applications can promote the selection of resistant pathogen populations and disrupt beneficial microbial and natural enemy communities, thereby compromising long-term control efficacy and increasing management costs [15]. In this context, the increasing incidence and severity of economically important plant diseases, together with the strong dependence on chemical pesticides, represent critical challenges for environmental sustainability at a global scale [16,17].

Biological control has emerged as a promising and more sustainable alternative for managing CLR, based on the use of living organisms or their metabolites through mechanisms such as parasitism, antibiosis, niche competition, and induction of plant resistance [18,19,20]. In response to the limitations of chemical control, interest has grown in the use of microorganisms and bioactive compounds with antifungal activity as alternative strategies against *H. vastatrix* [21]. Santiago et al. reported the existence of biological agents capable of inhibiting the pathogen or inducing systemic resistance in coffee crops, with laboratory and greenhouse conditions efficacies ranging from 50% to 100% [22]. However, despite promising results under controlled conditions, well-replicated field evaluations and multi-site validations remain comparatively limited, which constrains practical recommendations [23]. Species of the genus *Lecanicillium* have been reported to exhibit mycoparasitic activity against *H. vastatrix*, as evidenced by their colonization of rust sori shortly after inoculation [24]. Likewise, experimental studies have shown that *Lecanicillium* spp. can reduce urediniospore germination of the pathogen and decrease disease severity when applied before infection, highlighting their potential as biological control agents [25]. Additionally, isolates of *L. uredinophilum* have been documented to display hyperparasitic activity against urediniospores of *Phakopsora pachyrhizi*, the causal agent of Asian soybean rust, further reinforcing the mycoparasitic potential of the genus *Lecanicillium* against rust fungi [26].

The genus *Lecanicillium* has been extensively studied over the past decade through multilocus phylogenetic analyses based on markers such as LSU, TEF1, RPB1, and RPB2. Synthesis studies within the family Cordycipitaceae have established criteria of priority and monophyly, resolving nomenclatural duplications between sexual and asexual morphs and laying the foundations for the current circumscription of the genus [27].

Despite these advances, evidence specifically addressing the role of *L. uredinophilum* against *H. vastatrix* remains limited and dispersed across experimental and observational studies. This hinders a clear assessment of whether this fungus represents a robust biocontrol option relative to other agents previously evaluated for CLR management. Accordingly, this review addresses the following question: What is the potential of *L. uredinophilum* as a biocontrol agent of *H. vastatrix* compared with other previously evaluated species?

Accordingly, the objective of this review article is to critically analyze the available evidence on the potential of *L. uredinophilum* as a biocontrol agent against *H. vastatrix*, identifying the main advances, limitations, and research gaps, and evaluating its current status as an emerging biological agent from a comparative perspective grounded in the strength of existing scientific evidence.

## 2. Methodology

This review was conducted as a structured literature review guided by the general principles of the PRISMA 2020 framework to ensure a transparent and reproducible study identification and selection process [28]. The primary database search was performed on 15 January 2026 in Scopus and PubMed, selected for their broad coverage of peer-reviewed literature in plant pathology, microbiology, and biological control. The database search covered publications indexed between 2015 and January 2026 and used combinations of English keywords related to *Hemileia vastatrix*, coffee leaf rust, *Lecanicillium uredinophilum* (syn. *Akanthomyces uredinophilum*), mycoparasitism, antagonistic fungi, and biological control agents. Additional targeted terms associated with other fungal biocontrol agents, including *Lecanicillium lecanii* and *Trichoderma* spp., were included to support the comparative scope of the review. To complement the database search, relevant publications were also identified through manual screening of reference lists and citation tracking, enabling the incorporation of seminal studies published before 2015 that were directly relevant to the review objective.

A total of 162 records were initially identified through database searching, of which 2 were removed before eligibility assessment for reasons unrelated to duplication. Thirty-two reports were retrieved and assessed in full text, and 21 were excluded because they addressed different fungal species (*n* = 13) or were not sufficiently aligned with the main topic of the review (*n* = 8). Consequently, 24 studies from the database search met the eligibility criteria. An additional 76 relevant publications were incorporated through complementary sources, including citation tracking and targeted manual searches, resulting in a final corpus of 100 studies included in the qualitative synthesis. The selected literature was analyzed comparatively according to its relevance to the biology and epidemiology of *H. vastatrix*, mechanisms of fungal biocontrol, evidence related to *Lecanicillium* spp., specific information on *L. uredinophilum*, and comparisons with other biological control agents.

## 3. Integrated Analysis of the Evidence on Biological Control Agents

### 3.1. Coffee Leaf Rust (Hemileia vastatrix)

Understanding the epidemiology and infection biology of *H. vastatrix* is essential to identify critical stages susceptible to biological control interventions, particularly those involving mycoparasitic fungi. The epidemiology of *H. vastatrix* is strongly influenced by climatic factors, mainly temperature and precipitation. Maximum disease development usually occurs between October and March, under conditions of high humidity and temperatures ranging from 21.1 to 23.4 °C, following an annual cycle characterized by a slow growth phase (March–June), an accelerated phase (June–September), and an epidemic peak associated with increased inoculum dispersal [29]. Although *H. vastatrix* exhibits low genetic variability at the global scale, its genome, estimated at approximately 797 Mbp, harbors more than 50 physiological races and shows early activation of signaling and effector genes from germ tube formation, reflecting a highly specialized molecular interaction with its host [3]. In Peruvian coffee plantations, seven races of the pathogen have been identified, including two novel races, while Timor hybrid-derived varieties have shown persistent susceptibility, highlighting the pathogen’s adaptive capacity and the need for continuous monitoring before the introduction of new resistant varieties [30].

From a reproductive perspective, meiosis in the teliospores of *H. vastatrix* differs from that of most basidiomycetes, as the basidium is external and the chromosome number is higher; these irregularities observed under natural conditions suggest alterations during a critical phase of its life cycle [31]. The resulting basidiospores are not capable of infecting coffee plants, and no alternative host has been identified to date [3,32]. Consequently, urediniospores constitute the main unit of pathogen dissemination and reinfection and represent a critical target for mycoparasitic fungi capable of affecting their viability or germination.

**Figure 1:**
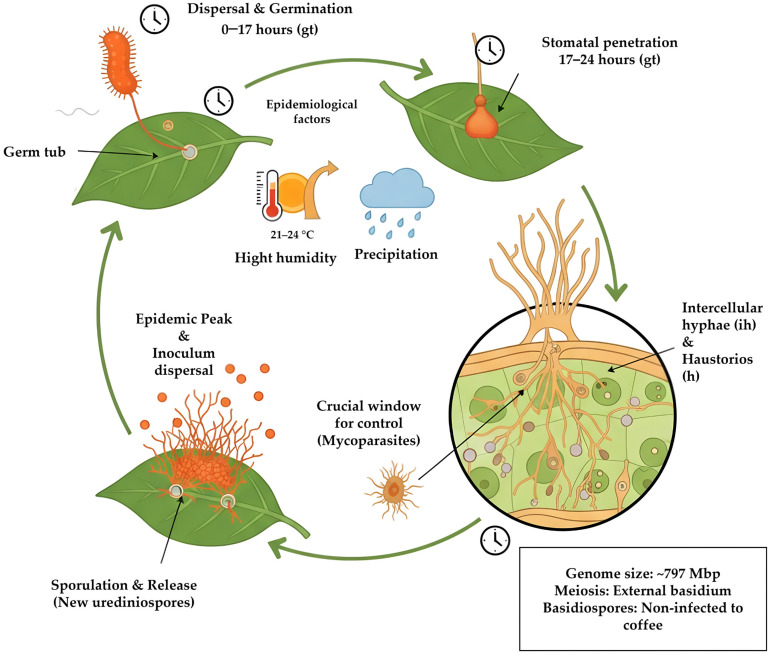
Infection process of *H. vastatrix*. Germinated urediniospore with germ tube and appressorium on coffee leaf stomata. Appressorium on stomata and penetration hypha (arrow). Appressorium on stomata and intercellular hyphae (ih) with haustorium (h) inside a subsidiary cell, LM. Intercellular hyphae (arrows) and haustoria (h) within epidermal and mesophyll cells. Haustorium within a spongy parenchyma cell. Intercellular hyphae (arrows) in the spongy parenchyma. Urediniosporic sorus protruding through stomata in a clustered form. Orange structures represent fungal structures, while green structures correspond to host plant tissues.

The infection process of *H. vastatrix* in coffee leaves, similar to that of other rust fungi, begins with adhesion to the host surface, followed by urediniospore germination, appressorium formation over stomata, penetration, and subsequent intercellular and intracellular colonization of plant tissues (Figure 1) [3,33].

During this process, the fungal cell wall, mainly composed of glucans and chitin, becomes particularly relevant from a biological control perspective because these structural polymers may serve as targets for hydrolytic enzymes produced by antagonistic fungi [34]. Haustorium formation is a key step in successful infection establishment, as these structures facilitate nutrient exchange between the pathogen and the host [35]. At advanced infection stages, haustoria become encapsulated by material reacting positively to compounds such as callose and glucans, which has been interpreted as a plant defense response; however, this reaction occurs too late to prevent fungal growth and sporulation [36].

### 3.2. Biological Control of Phytopathogenic Fungi

Biological control of plant diseases is based on the use of living organisms or their metabolites through mechanisms such as parasitism, antibiosis, niche competition, and induction of plant resistance [18,19]. Its implementation in disease management programs requires careful evaluation, as efficacy often differs between controlled and field conditions. In this context, biological control represents a sustainable alternative to chemical fungicides by reducing chemical residues and toxicological risks [20]. Among fungal biocontrol agents, *Trichoderma* spp. are widely recognized as a model genus due to their ability to integrate multiple mechanisms of action, including mycoparasitism, antibiosis, competition, and induction of plant defense responses [37,38,39]. However, variability in performance under field conditions highlights the need for a deeper mechanistic understanding of these interactions [40,41,42].

In fungal antagonists, these mechanisms operate as part of an integrated sequence involving host recognition, adhesion, and penetration of the pathogen, followed by degradation of fungal structures. This process is mediated by extracellular hydrolytic enzymes such as chitinases, β-1,3-glucanases, proteases, and N-acetylglucosaminidases, which target key components of fungal cell walls and interfere with spore viability, germination, and early infection stages [43,44,45]. Antibiosis further contributes through the production of secondary metabolites with antifungal activity, including gliotoxin, gliovirin, peptaibols (e.g., alamethicin), 6-pentyl-2H-pyran-2-one (6-PP), harzianic acid, and harzianolide, which can disrupt membrane integrity, induce oxidative stress, and inhibit fungal growth [45,46,47]. These mechanisms often act synergistically, enhancing overall antagonistic effectiveness [44,46].

Competition for nutrients and space is particularly relevant on plant surfaces, where environmental constraints such as UV radiation, fluctuating humidity, and nutrient limitation influence microbial establishment and persistence [43]. In addition, some fungal biocontrol agents interact with plant tissues as endophytes or rhizosphere-associated microorganisms, inducing systemic resistance through signaling pathways commonly associated with jasmonic acid and ethylene, and in some cases salicylic acid [48]. These responses involve activation of defense-related genes, reactive oxygen species production, and structural barriers such as callose deposition, contributing to reduced disease development [49,50,51].

### 3.3. The Genus Lecanicillium spp.

Recent phylogenetic studies analyzing the *Akanthomyces sensu lato* complex have demonstrated that it comprises several independent lineages within the family Cordycipitaceae. These analyses have led to taxonomic reorganization, including the redefinition of *Lecanicillium* for specific clades, to better reflect evolutionary relationships among these fungi [52]. Representative species such as *Lecanicillium muscarium* (formerly *Akanthomyces muscarius*) have shown dual biocontrol potential, combining entomopathogenic activity against insect pests with antagonistic effects against phytopathogenic fungi, supporting their relevance in integrated pest and disease management strategies [53].

Consistent with the general mechanisms described above, *Lecanicillium* spp. comprise fungi with broad potential as biological control agents. Their mode of action involves both mechanical penetration and the production of hydrolytic enzymes, enabling them to infect insect hosts or parasitize other fungi. These mechanisms may operate in conjunction with the induction of systemic resistance responses in plants, highlighting their multifunctional role in plant protection [54]. This interaction typically follows a sequential process involving adhesion to the host surface, germination, localized penetration, and enzymatic degradation of structural components.

Hydrolytic enzymes such as chitinases, β-1,3-glucanases, and proteases play a central role in this process by specifically targeting structural polymers of fungal cell walls, including chitin and glucans. This enzymatic activity weakens cell wall integrity, facilitating penetration and leading to lysis of infective structures such as urediniospores, thereby directly impairing pathogen viability and infection capacity [55,56].

In addition to enzymatic degradation, *Lecanicillium* spp. may produce secondary metabolites that contribute to antibiosis. Although less characterized than in *Trichoderma*, metabolite profiling studies indicate that species within the genus can synthesize bioactive compounds, including cyclic depsipeptides and other low-molecular-weight metabolites with antimicrobial properties [57,58]. These compounds may interfere with spore germination, membrane integrity, or hyphal development, reinforcing antagonistic activity.

Beyond direct antagonism, some species of *Lecanicillium* may also interact with plant tissues as endophytes or epiphytes, expanding their functional role in plant protection systems [54,59]. In this context, their contribution to disease suppression may include mechanisms consistent with induced systemic resistance, as described for other fungal biocontrol agents, potentially enhancing the plant’s ability to restrict pathogen establishment during early infection stages. However, compared to direct mycoparasitism, this indirect mechanism remains less well characterized in coffee leaf rust.

Species of the genus *Lecanicillium* are primarily recognized as entomopathogenic fungi; however, their association with CLR has also been documented. Observational studies conducted in coffee plantations in Indonesia reported the presence of *Lecanicillium* spp. associated with *H. vastatrix* infections, suggesting a potential ecological interaction under natural conditions [60].

**Figure 2:**
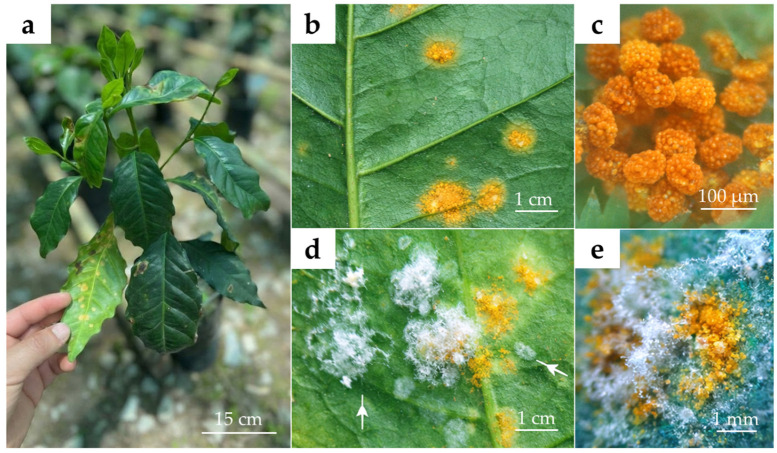
Interaction between *Lecanicillium* spp. and coffee leaf rust (*Hemileia vastatrix*). (**a**) Symptomatic coffee plant under nursery conditions. (**b**) Uredinia of *H. vastatrix* on the abaxial leaf surface. (**c**) Uredinial structures of *H. vastatrix*. (**d**) Hyperparasitic colonization of rust pustules by *Lecanicillium* spp.; white arrows indicate areas of fungal colonization. (**e**) Stereomicroscopic detail of partially colonized uredinia.

Evidence from field observations indicates that fungi belonging to the *Lecanicillium lecanii* complex are frequently found colonizing rust lesions, where they reduce urediniospore viability and suggest the occurrence of natural mycoparasitism in coffee agroecosystems [61,62,63] (Figure 2). At the plot level, increased incidence of these fungi has been associated with reduced urediniospore germination and visible colonization of rust pustules [64]. In agreement with these findings, *L. uredinophilum* was originally isolated from rust sori and described as a fungicolous species associated with uredinal fungi, providing direct evidence of its interaction with *H. vastatrix* [65].

Experimental evidence supports these field observations. *Lecanicillium* spp. have been reported to exhibit mycoparasitism on *H. vastatrix*, reaching up to 68% parasitism of urediniospores at 120 h post-inoculation, although further validation under field conditions is required [24]. Similarly, controlled assays have shown that *L. psalliotae* significantly reduces urediniospore germination, while its own germination increases in the presence of the pathogen. Under in vivo conditions, application before pathogen inoculation resulted in the greatest reduction in disease severity, indicating a preventive effect [25]. Additional studies evaluating multiple *Lecanicillium* isolates have revealed variability in mycoparasitic capacity; nevertheless, several strains were able to penetrate and degrade urediniospores effectively, supporting the potential of the genus as a biological control agent against CLR [66].

The accumulation of evidence from field studies, laboratory assays, and molecular analyses indicates that species of the genus *Lecanicillium* are plausible mycoparasites of *H. vastatrix* and represent promising candidates for biological control strategies. However, current evidence remains limited in terms of consistency, field validation, and mechanistic understanding, highlighting the need for further research focused on efficacy under field conditions, strain selection, and formulation development [57,58,66,67,68].

### 3.4. Mycoparasitic Activity of Lecanicillium uredinophilum

The species was first described as *L. uredinophilum* based on isolates obtained from rust pustules in Korea; colonial morphology allowed its diagnosis as a new fungicolous species associated with fungi of the order Uredinales [65]. In culture, colonies typically exhibit a whitish appearance, with mycelium partially immersed in the host sori and hyaline conidia of cylindrical to oblong or narrowly ellipsoid shape, traits that have been confirmed both in the original description and in subsequent morphological revisions [65].

Although *L. uredinophilum* was initially reported as a mycoparasite of urediniospores, later studies have shown that genetically equivalent strains can also be isolated from insects, suggesting a broad ecological range that includes both fungicolous and entomopathogenic forms [69]. Experimental assays have indicated optimal growth temperatures around 21–24 °C and the ability to develop on standard culture media, information that is relevant for the production and formulation of experimental strains. In addition, its metabolite production and insecticidal activity have been explored in bioassays against various pests, revealing additional applied potential [70].

**Figure 3:**
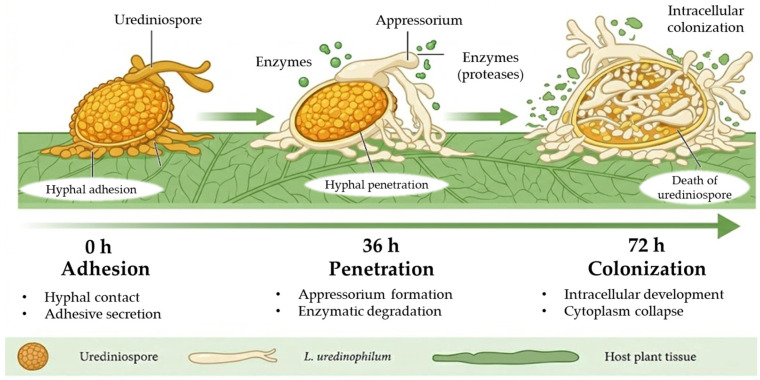
Proposed mechanism of mycoparasitism by *Lecanicillium uredinophilum* on rust urediniospores.

*L. uredinophilum* exhibits a high mycoparasitic capacity against *P. pachyrhizi,* showing through confocal microscopy and electron microscopy the active penetration and colonization of urediniospores within approximately 36 h, accompanied by degradation of the cell wall and collapse of internal structures [26]. These findings confirm a mode of action based on direct mycoparasitism and support its high potential as a biocontrol agent of Asian soybean rust [24]. A schematic representation of this proposed mycoparasitic mechanism is shown in Figure 3.

From a broader ecological perspective, some microorganisms can temporarily inhabit plant tissues without causing disease symptoms and that, in many cases, provides benefits to the host plant [71]. The concept of endophytic fungi was introduced by De Bary in 1866, who initially described them as neutral organisms with no evident harmful or beneficial effects on their plant hosts [72]. In the case of *L. uredinophilum*, studies on its endophytic behavior in coffee remain limited; however, assays conducted on coffee cv. Caturra showed that three out of fourteen evaluated strains achieved high levels of internal colonization, and two of them stood out for their endophytic stability, demonstrating a persistent interaction with the host. Although species such as *Trichoderma* promoted plant growth to a greater extent, *L. uredinophilum* was notable for its capacity for internal colonization and stability, traits relevant to biological control [73]. Nevertheless, these studies did not assess its direct action against *H. vastatrix*, and therefore, an information gap persists regarding its specific efficacy as a biocontrol agent of CLR, highlighting the need for further research aimed at determining whether its endophytic colonization can be translated into antagonistic mechanisms or the induction of resistance against this highly relevant pathogen in coffee production.

### 3.5. Comparison with Other Biocontrol Agents for Coffee Leaf Rust

Recent literature reports a broad spectrum of microbial agents with biocontrol potential against *H. vastatrix*, including mycoparasitic fungi, such as *Trichoderma* spp., *Calonectria hemileiae*, and *Lecanicillium*/*Akanthomyces*-like taxa, as well as antagonistic bacteria belonging mainly to *Bacillus*, *Pseudomonas*, and *Paenibacillus*. Their efficacy is highly context-dependent, being influenced by strain identity, formulation, application timing, and environmental conditions; therefore, most strategies emphasize their integration within broader coffee leaf rust (CLR) management programs [3,22,74,75].

Among fungal agents, *Trichoderma* spp. are the most extensively studied and operationally versatile, combining multiple mechanisms such as mycoparasitism, antibiosis, competition, and induction of plant defenses, along with reported association with rust pustules and endophytic colonization of *Coffea* tissues [76,77]. Although reductions in disease severity have been documented, their effectiveness remains variable and dependent on local conditions and application strategies [78,79].

In contrast, *Calonectria hemileiae* exhibits a more specialized interaction with CLR, including strong inhibition of urediniospore germination, confirmation of mycoparasitism through Koch’s postulates, and reductions in disease severity of up to 70–90% under controlled conditions [80]. Additionally, its activity has been associated with increased plant defense responses. Nevertheless, evidence remains largely confined to controlled environments, limiting its field-level validation (Table 1).

The *Lecanicillium* complex shows greater variability. Previous and recent studies have reported hyperparasitism of coffee rust and identified *Lecanicillium*-like taxa, including *L. uredinophilum*, associated with *H. vastatrix* [63,65,81]. Despite its biological plausibility, *L. uredinophilum* remains less validated than other fungal agents, with evidence still limited in scope and scale.

Bacterial antagonists provide a complementary perspective. Field studies have shown that *Bacillus* isolates can achieve reductions in rust intensity comparable to copper-based treatments, while *Pseudomonas* shows intermediate effects [75,82]. More recently, *Paenibacillus* sp. demonstrated high efficacy under greenhouse conditions, significantly reducing urediniospore germination, incidence, and severity [21]. These findings suggest that bacterial agents may offer effective preventive strategies, although environmental and operational factors also influence their performance.

### 3.6. Current Limitations

Despite multiple records documenting the colonization of rust sori by *L. uredinophilum* [26,65,69], direct, reproducible, and conclusive evidence supporting its efficacy against *H. vastatrix* under field conditions remains scarce. Most available information derives from laboratory assays or isolated observations, which do not fully capture the complexity of ecological interactions present in commercial coffee plantations. Environmental variability, microclimatic conditions, pathogen genetic diversity, and interactions with other associated organisms may significantly influence the effectiveness of this fungus, highlighting the need for controlled, replicated, multi-location trials to validate its performance under real agricultural conditions. In contrast, other microbial agents for CLR, including *Trichoderma* spp. and bacterial antagonists such as *Bacillus* and *Paenibacillus*, have been evaluated under greenhouse or field-oriented conditions [21,79]. Additionally, entomopathogenic fungi such as *Beauveria bassiana* have demonstrated the ability to establish as endophytes in coffee tissues, supporting the broader concept that fungal colonization of plant tissues may be achievable, although this does not constitute direct evidence of CLR control by *L. uredinophilum* [84].

In addition to the limited field evidence, important constraints remain regarding inoculant production and formulation. The availability of stable *L. uredinophilum* inoculants is currently restricted, as most studies have been conducted at laboratory or small experimental scales without standardized protocols for mass production [66,67]. Challenges related to long-term viability, tolerance to storage conditions, and maintenance of biocontrol activity after application further complicate its practical use. Moreover, the absence of optimized application strategies, such as dosage, frequency, and delivery methods limits its integration into commercial production systems. These limitations are consistent with broader challenges reported for microbial biocontrol agents, whose performance is often affected by environmental factors such as UV radiation, temperature, and humidity [85,86].

Another critical limitation is the lack of information regarding the interaction of *L. uredinophilum* with common agronomic practices, including fertilization, pruning, agrochemical use, irrigation, and pest management. These practices can alter foliar microflora, surface moisture, and the microclimate surrounding leaves, thereby influencing fungal colonization, persistence, and efficacy on *H. vastatrix* sori. Consequently, evaluating the compatibility of this biocontrol agent within real production systems is essential to determine its practical applicability [86,87,88].

Finally, gaps remain in genomic and functional characterization. Although studies in related species have identified genes associated with lytic enzymes and secondary metabolites with biocontrol potential [89], specific functional analyses for *L. uredinophilum* are still lacking to directly link these molecular determinants with its mycoparasitic activity against *H. vastatrix*. This limitation is particularly relevant given its ecological versatility and ongoing taxonomic refinement within *Lecanicillium*/*Akanthomyces*-like taxa, which may obscure strain-level functional differences [69,90]. Addressing these gaps will be essential for improving strain selection and developing more effective and biologically consistent formulations.

## 4. Critical Discussion of the Biocontrol Potential of *L. uredinophilum* Against *H. vastatrix*

The evidence analyzed in this review suggests that *L. uredinophilum* should be interpreted as a biologically coherent candidate, yet still at an early stage of technological development and insufficiently validated under field conditions for the management of coffee leaf rust (CLR). Its main strength lies in its affinity for rust fungi, particularly its ability to associate with uredinial structures and parasitize urediniospores, which are key elements in the dispersal and reinfection cycle of *H. vastatrix* [24,27,61]. This correspondence with the pathogen’s biology supports its functional plausibility. However, this biological coherence contrasts with a still limited experimental basis, largely supported by studies conducted under controlled conditions and extrapolations from related species rather than robust field-based evidence [24,63].

The dependence of *H. vastatrix* on viable urediniospores for dissemination and reinfection reinforces the relevance of strategies targeting these structures. The infection process involves specialized structures such as appressoria and haustoria, as well as intercellular development closely associated with host foliar tissues, making urediniospores a critical point in the pathogen’s life cycle [3,30,91]. In this context, the ability of *L. uredinophilum* to establish direct contact with these structures is consistent with a mode of action targeting early and highly vulnerable stages of infection.

When compared with other microbial agents evaluated for CLR management, its position becomes clearer. Species of the genus *Trichoderma* exhibit a clear advantage in terms of mechanistic versatility and experimental validation, supported by the integration of multiple modes of action, including mycoparasitism, antibiosis, competition for resources, and plant growth promotion [76]. Similarly, bacterial genera such as *Bacillus* and *Paenibacillus* have demonstrated consistent performance through the production of antifungal lipopeptides, inhibition of urediniospore germination, and induction of systemic resistance in the host [64,92]. In particular, *Paenibacillus* sp. has been reported to significantly reduce urediniospore germination, disease incidence, and severity of CLR under experimental conditions, indicating that some bacterial agents have already reached a functional level within the CLR pathosystem [21].

Against this background, *L. uredinophilum* appears to occupy a more specialized niche, characterized by direct interaction with pathogen structures. This specialization may confer an advantage in terms of biological precision; however, it also implies lower versatility and a more limited body of evidence, which currently restricts its competitiveness relative to more established, generalist agents. This difference becomes even more evident when compared with specialized fungal antagonists such as *Calonectria hemileiae*, which has demonstrated reductions in rust severity of up to 70–90% under experimental conditions [80]. These contrasts indicate that the main limitation of *L. uredinophilum* is primarily translational rather than conceptual, as its biological relevance is evident but not yet supported by sufficient validation for practical implementation [93].

The mechanisms described for species of the genus *Lecanicillium* are primarily associated with direct mycoparasitism, mediated by extracellular hydrolytic enzymes such as chitinases, β-1,3-glucanases, and proteases, which target the structural components of fungal cell walls [25,94]. Evidence from related species indicates that these enzymatic systems play a decisive role in the degradation of urediniospores and in disrupting the infection process, supporting the plausibility of a similar mechanism in *L. uredinophilum* [95]. These enzymes likely compromise cell wall integrity, leading to reduced spore viability and interference with germination and penetration processes, which are critical for successful host colonization.

The potential role of antifungal secondary metabolites adds another relevant dimension. In *Bacillus*, compounds such as surfactins, iturins, and fengycins act directly on fungal membrane integrity [92], while in *Trichoderma*, these metabolites complement mycoparasitic and competitive interactions [76,96]. In the case of *L. uredinophilum*, however, information on the production and function of such compounds remains limited, representing a major mechanistic gap. These metabolites may interfere with membrane stability or early hyphal development, potentially acting in synergy with enzymatic mechanisms, although their role in this specific pathosystem remains unclear [92]. Additionally, endophytic colonization has been reported in species of the genus *Lecanicillium*; its contribution to CLR suppression through induced resistance or modification of the foliar microenvironment has not been conclusively demonstrated [97]. Consequently, direct mycoparasitism remains the most strongly supported mechanism, while indirect effects remain hypothetical.

A major limitation for the validation of *L. uredinophilum* as a biological control agent is the scarcity of field-based studies. Most of the available evidence derives from laboratory or greenhouse experiments, making it difficult to assess its stability, persistence, and efficacy under the environmental variability characteristic of coffee-growing systems. Factors such as UV radiation, temperature, humidity, and interactions with native microbiota can significantly influence both rust development and the performance of microbial antagonists [30,98].

Advancing *L. uredinophilum* toward practical applications requires a research agenda focused on addressing these gaps. Priority should be given to multi-site field trials, deeper molecular and biochemical characterization of its interaction with *H. vastatrix*, and the integration of omics approaches to identify key genes, enzymes, and metabolites [99]. Furthermore, evaluating intraspecific variability will be essential to identify strains with higher biotechnological potential in terms of efficacy, stability, and environmental adaptability.

The transition from an experimental candidate to a functional biopesticide also involves challenges related to mass production, formulation development, product stability, and shelf life [43,100]. In addition, regulatory frameworks require rigorous assessments of safety, environmental impact, and effects on non-target organisms, aspects that have not yet been specifically addressed for this species [43].

## 5. Conclusions

The available evidence indicates that *L. uredinophilum* exhibits a consistent and biologically coherent mycoparasitic potential against rust fungi, particularly through its ability to target urediniospores, a critical component in the infection cycle of *H. vastatrix*. This functional alignment supports its consideration as an emerging candidate for the biological control of coffee leaf rust.

However, despite this promising biological foundation, the current level of evidence remains insufficient to support its practical implementation. Most studies have been conducted under controlled conditions, and key aspects such as field efficacy, environmental stability, formulation development, and regulatory evaluation remain largely unexplored. In comparison with more established biocontrol agents, *L. uredinophilum* is still positioned at an early stage of technological development.

Future progress will depend on the generation of robust field-based evidence, the clarification of its mechanisms of action beyond mycoparasitism, and its successful integration into viable formulations compatible with integrated disease management strategies. Thus, rather than being considered a consolidated solution, *L. uredinophilum* should be viewed as a promising but still developing biological agent whose practical relevance will ultimately depend on its successful translation from experimental systems to real agricultural conditions.

## Figures and Tables

**Table 1:** Comparative overview of biological control agents evaluated against CLR.

Biocontrol Agent/Group	Representative Taxa/Strains	Main Mechanisms	Evidence Against CLR	Comparative Interpretation	Representative References
*Trichoderma* spp.	*T. harzianum*, *T. asperellum*, consortia	Mycoparasitism, antibiosis, competition, induced resistance, endophytism	Association with rust pustules; field reductions in severity, stronger in preventive applications	Most versatile and operationally mature fungal platform; performance remains context-dependent	[22,76,79]
*Calonectria hemileiae*	*C. hemileiae*	Specialized mycoparasitism; inhibition of spore germination; host defense activation	>80% inhibition of germination; 70–90% reduction in severity (controlled conditions)	Strongest specialized fungal antagonist under controlled conditions; limited field validation	[11,22,80]
*Lecanicillium*/*Akanthomyces*-like taxa	*L. lecanii*, *L. uredinophilum*	Hyperparasitism of sori and urediniospores; possible ecological/endophytic effects	Recurrent detection in CLR systems; hyperparasitism documented; limited field-scale validation	Rust-oriented but heterogeneous group; less mature than *Trichoderma*; *L. uredinophilum* remains in the early stage	[24,63,64,65,81]
*Bacillus* spp.	*Bacillus* sp. B157, *B. subtilis*	Antibiosis, lipopeptides, induced resistance	Field performance comparable to copper; reduction in incidence and spore growth	Most consistent bacterial option; still strain- and context-dependent	[22,75,82,83]
*Pseudomonas* spp.	*Pseudomonas* sp. P286, *P. fluorescens*	Antibiosis, competition and induced resistance	Moderate field performance; inhibition of urediniospore growth	Secondary bacterial option; weaker evidence than *Bacillus*	[22,75,82]
*Paenibacillus* spp.	*Paenibacillus* sp. NMA1017	Antibiosis; inhibition of germination	Up to 94% germination inhibition; strong reductions in incidence and severity (greenhouse)	Promising recent candidate; requires independent and field validation	[11,21,22]

## Data Availability

No new data were created or analyzed in this study. All data discussed are derived from previously published studies, which are cited within the article. Data sharing is therefore not applicable.

## References

[1] Wright D.R., Bekessy S.A., Lentini P.E., Garrard G.E., Gordon A., Rodewald A.D., Bennett R.E., Selinske M.J. (2024). Sustainable Coffee: A Review of the Diverse Initiatives and Governance Dimensions of Global Coffee Supply Chains. Ambio.

[2] Adhikari M., Isaac E.L., Paterson R.R.M., Maslin M.A. (2020). A Review of Potential Impacts of Climate Change on Coffee Cultivation and Mycotoxigenic Fungi. Microorganisms.

[3] Talhinhas P., Batista D., Diniz I., Vieira A., Silva D.N., Loureiro A., Tavares S., Pereira A.P., Azinheira H.G., Guerra-Guimarães L. (2017). The Coffee Leaf Rust Pathogen *Hemileia vastatrix*: One and a Half Centuries around the Tropics. Mol. Plant Pathol..

[4] Freitas V.V., Borges L.L.R., Vidigal M.C.T.R., Dos Santos M.H., Stringheta P.C. (2024). Coffee: A Comprehensive Overview of Origin, Market, and the Quality Process. Trends Food Sci. Technol..

[5] International Coffee Organization (ICO) Coffee Market Report—Statistics Section. https://ico.org/es/resources/coffee-market-report-statistics-section/.

[6] Camara Peruana de Cafe y Cacao El Consumidor de Café Peruano. https://camcafeperu.com.pe/ES/cafe-peruano-destacados-nota.php?id=2.

[7] Savary S., Willocquet L., Pethybridge S.J., Esker P., McRoberts N., Nelson A. (2019). The Global Burden of Pathogens and Pests on Major Food Crops. Nat. Ecol. Evol..

[8] Bebber D.P., Castillo Á.D., Gurr S.J. (2016). Modelling Coffee Leaf Rust Risk in Colombia with Climate Reanalysis Data. Philos. Trans. R. Soc. Lond. B Biol. Sci..

[9] McCook S. (2006). Global Rust Belt: *Hemileia vastatrix* and the Ecological Integration of World Coffee Production Since 1850. J. Glob. Hist..

[10] Koutouleas A. (2023). Coffee Leaf Rust: Wreaking Havoc in Coffee Production Areas Across the Tropics. Plant Health Cases.

[11] Salazar Navarro A., Ruíz-Valdiviezo V., Joya Dávila G., Gonzalez-Mendoza D. (2024). Coffee Leaf Rust (*Hemileia vastatrix*) Disease in Coffee Plants and Perspectives by the Disease Control. Phyton-Int. J. Exp. Bot..

[12] Kilambo D., Reuben S., Mamiro D. (2013). Responses of Compact Coffee Clones Against Coffee Berry and Coffee Leaf Rust Diseases in Tanzania. J. Plant Stud..

[13] Loland J.Ø., Singh B.R. (2004). Copper Contamination of Soil and Vegetation in Coffee Orchards after Long Use of Cu Fungicides. Nutr. Cycl. Agroecosyst..

[14] Gilden R.C., Huffling K., Sattler B. (2010). Pesticides and Health Risks. J. Obstet. Gynecol. Neonatal Nurs. JOGNN.

[15] Lamichhane J.R., Osdaghi E., Behlau F., Köhl J., Jones J.B., Aubertot J.-N. (2018). Thirteen Decades of Antimicrobial Copper Compounds Applied in Agriculture. A Review. Agron. Sustain. Dev..

[16] Fones H.N., Bebber D.P., Chaloner T.M., Kay W.T., Steinberg G., Gurr S.J. (2020). Threats to Global Food Security from Emerging Fungal and Oomycete Crop Pathogens. Nat. Food.

[17] El-Saadony M.T., Saad A.M., Soliman S.M., Salem H.M., Ahmed A.I., Mahmood M., El-Tahan A.M., Ebrahim A.A.M., Abd El-Mageed T.A., Negm S.H. (2022). Plant Growth-Promoting Microorganisms as Biocontrol Agents of Plant Diseases: Mechanisms, Challenges and Future Perspectives. Front. Plant Sci..

[18] Lahlali R., Ezrari S., Radouane N., Kenfaoui J., Esmaeel Q., El Hamss H., Belabess Z., Barka E.A. (2022). Biological Control of Plant Pathogens: A Global Perspective. Microorganisms.

[19] Pandit M.A., Kumar J., Gulati S., Bhandari N., Mehta P., Katyal R., Rawat C.D., Mishra V., Kaur J. (2022). Major Biological Control Strategies for Plant Pathogens. Pathogens.

[20] He D.-C., He M.-H., Amalin D.M., Liu W., Alvindia D.G., Zhan J. (2021). Biological Control of Plant Diseases: An Evolutionary and Eco-Economic Consideration. Pathogens.

[21] Gómez-de la Cruz I., Martínez-Bolaños M., Chávez-Ramírez B., Estrada-de los Santos P. (2024). Biocontrol of *Hemileia vastatrix*, the Causal Agent of Coffee Leaf Rust, by *Paenibacillus* sp. NMA1017. Plant Dis..

[22] Santiago-Santiago M., Sánchez-Viveros G., Hernández-Adame L., Chiquito-Contreras C.J., Salinas-Castro A., Chiquito-Contreras R.G., Hernández-Montiel L.G. (2023). Essential Oils and Antagonistic Microorganisms as Eco-Friendly Alternatives for Coffee Leaf Rust Control. Plants.

[23] Hernández C., López Escobar L., Sánchez-Tafolla L. (2021). Agentes de Control Biológico de La Roya Del Café ¿Cómo Funcionan y Qué Tan Efectivos Son?. BioTecnología.

[24] Gómez-De La Cruz I., Pérez-Portilla E., Escamilla-Prado E., Martínez-Bolaños M., Carrión-Villarnovo G.L.L., Hernández-Leal T.I. (2018). Selección in vitro de micoparásitos con potencial de control biológico sobre roya del café (*Hemileia vastatrix*). Rev. Mex. Fitopatol..

[25] Mahfud M.C., Mior Ahmad Z.A., Meon S., Kadir J. (2006). In Vitro and In Vivo Tests for Parasitism of *Verticillium psalliotae* Treschow on *Hemileia vastatrix* BERK. and BR. Malays. J. Microbiol..

[26] Mwelasi P.P., Laing M.D., Ibaba J.D., Rogers R., Németh M.Z., Yobo K.S. (2025). Ultrastructural Examination of the Fungus-To-Fungus Interactions of *Lecanicillium uredinophilum* and *Phakopsora pachyrhizi*. Plant-Environ. Interact..

[27] Kepler R.M., Luangsa-ard J.J., Hywel-Jones N.L., Quandt C.A., Sung G.-H., Rehner S.A., Aime M.C., Henkel T.W., Sanjuan T., Zare R. (2017). A Phylogenetically-Based Nomenclature for Cordycipitaceae (Hypocreales). IMA Fungus.

[28] Haddaway N.R., Page M.J., Pritchard C.C., McGuinness L.A. (2022). PRISMA2020: An R Package and Shiny App for Producing PRISMA 2020-Compliant Flow Diagrams, with Interactivity for Optimised Digital Transparency and Open Synthesis. Campbell Syst. Rev..

[29] Araño-Leyva L., Prieto-García D., Rodríguez-Patterson F. (2017). Situación epidemiológica de la roya del cafeto (*Hemileia vastatrix* Berkeley & Broome) en las condiciones agroecológicas de Tercer Frente, Cuba. Café Cacao.

[30] Julca-Otiniano A., Alvarado-Huamán L., Castro-Cepero V., Borjas-Ventura R., Gómez-Pando L., Pereira A.P., Nielen S., Ingelbrecht I., Silva M.d.C., Várzea V. (2024). New Races of *Hemileia vastatrix* Detected in Peruvian Coffee Fields. Agronomy.

[31] Chinnappa C., Sreenivasan M. (1965). Cytological Studies on Germinating Teliospores of *Hemileia vastatrix*. Caryologia.

[32] Kushalappa A.C., Eskes A.B. (1989). Advances in Coffee Rust Research. Annu. Rev. Phytopathol..

[33] Assante G., Maffi D., Saracchi M., Farina G., Moricca S., Ragazzi A. (2004). Histological Studies on the Mycoparasitism of *Cladosporium tenuissimum* on Urediniospores of *Uromyees appendiculatus*. Mycol. Res..

[34] Karlsson M., Atanasova L., Jensen D.F., Zeilinger S. (2017). Necrotrophic Mycoparasites and Their Genomes. Microbiol. Spectr..

[35] Mapuranga J., Zhang L., Zhang N., Yang W. (2022). The Haustorium: The Root of Biotrophic Fungal Pathogens. Front. Plant Sci..

[36] Silva M.C., Nicole M., Rijo L., Geiger J.P., Rodrigues C.J. (1999). Cytochemical Aspects of the Plant–Rust Fungus Interface during the Compatible Interaction *Coffea arabica* (Cv. Caturra)–*Hemileia vastatrix* (Race III). Int. J. Plant Sci..

[37] Yao X., Guo H., Zhang K., Zhao M., Ruan J., Chen J. (2023). *Trichoderma* and Its Role in Biological Control of Plant Fungal and Nematode Disease. Front. Microbiol..

[38] Keswani C., Mishra S., Sarma B.K., Singh S.P., Singh H.B. (2014). Unraveling the Efficient Applications of Secondary Metabolites of Various *Trichoderma* Spp. Appl. Microbiol. Biotechnol..

[39] Dutta P., Mahanta M., Singh S.B., Thakuria D., Deb L., Kumari A., Upamanya G.K., Boruah S., Dey U., Mishra A.K. (2023). Molecular Interaction between Plants and *Trichoderma* Species against Soil-Borne Plant Pathogens. Front. Plant Sci..

[40] Alfiky A., Weisskopf L. (2021). Deciphering *Trichoderma*–Plant–Pathogen Interactions for Better Development of Biocontrol Applications. J. Fungi.

[41] Spadaro D., Droby S. (2016). Development of Biocontrol Products for Postharvest Diseases of Fruit: The Importance of Elucidating the Mechanisms of Action of Yeast Antagonists. Trends Food Sci. Technol..

[42] Freimoser F.M., Rueda-Mejia M.P., Tilocca B., Migheli Q. (2019). Biocontrol Yeasts: Mechanisms and Applications. World J. Microbiol. Biotechnol..

[43] Köhl J., Kolnaar R., Ravensberg W.J. (2019). Mode of Action of Microbial Biological Control Agents Against Plant Diseases: Relevance Beyond Efficacy. Front. Plant Sci..

[44] Kubicek C.P., Herrera-Estrella A., Seidl-Seiboth V., Martinez D.A., Druzhinina I.S., Thon M., Zeilinger S., Casas-Flores S., Horwitz B.A., Mukherjee P.K. (2011). Comparative Genome Sequence Analysis Underscores Mycoparasitism as the Ancestral Life Style of *Trichoderma*. Genome Biol..

[45] Sood M., Kapoor D., Kumar V., Sheteiwy M.S., Ramakrishnan M., Landi M., Araniti F., Sharma A. (2020). *Trichoderma*: The “Secrets” of a Multitalented Biocontrol Agent. Plants.

[46] Vinale F., Sivasithamparam K., Ghisalberti E.L., Marra R., Woo S.L., Lorito M. (2008). *Trichoderma*–Plant–Pathogen Interactions. Soil Biol. Biochem..

[47] Mendoza-Mendoza A., Esquivel-Naranjo E.U., Soth S., Whelan H., Alizadeh H., Echaide-Aquino J.F., Kandula D., Hampton J.G. (2024). Uncovering the Multifaceted Properties of 6-Pentyl-Alpha-Pyrone for Control of Plant Pathogens. Front. Plant Sci..

[48] Shoresh M., Yedidia I., Chet I. (2005). Involvement of Jasmonic Acid/Ethylene Signaling Pathway in the Systemic Resistance Induced in Cucumber by *Trichoderma asperellum* T203. Phytopathology.

[49] Martínez-Medina A., Fernández I., Sánchez-Guzmán M.J., Jung S.C., Pascual J.A., Pozo M.J. (2013). Deciphering the Hormonal Signalling Network behind the Systemic Resistance Induced by *Trichoderma harzianum* in Tomato. Front. Plant Sci..

[50] Yu Y., Gui Y., Li Z., Jiang C., Guo J., Niu D. (2022). Induced Systemic Resistance for Improving Plant Immunity by Beneficial Microbes. Plants.

[51] Mengistu A.A. (2020). Endophytes: Colonization, Behaviour, and Their Role in Defense Mechanism. Int. J. Microbiol..

[52] Khonsanit A., Thanakitpipattana D., Mongkolsamrit S., Kobmoo N., Phosrithong N., Samson R.A., Crous P.W., Luangsa-Ard J.J. (2024). A Phylogenetic Assessment of *Akanthomyces sensu lato* in *Cordycipitaceae* (*Hypocreales*, *Sordariomycetes*): Introduction of New Genera, and the Resurrection of *Lecanicillium*. Fungal Syst. Evol..

[53] Saidi A., Mebdoua S., Mecelem D., Al-Hoshani N., Sadrati N., Boufahja F., Bendif H. (2023). Dual Biocontrol Potential of the Entomopathogenic Fungus *Akanthomyces muscarius* against *Thaumetopoea pityocampa* and Plant Pathogenic Fungi. Saudi J. Biol. Sci..

[54] Goettel M.S., Koike M., Kim J.J., Aiuchi D., Shinya R., Brodeur J. (2008). Potential of *Lecanicillium* spp. for Management of Insects, Nematodes and Plant Diseases. J. Invertebr. Pathol..

[55] Gan Z., Yang J., Tao N., Liang L., Mi Q., Li J., Zhang K.-Q. (2007). Cloning of the Gene *Lecanicillium psalliotae* Chitinase Lpchi1 and Identification of Its Potential Role in the Biocontrol of Root-Knot Nematode Meloidogyne Incognita. Appl. Microbiol. Biotechnol..

[56] Nguyen H., Quyen D., Nguyen S., Vu V. (2015). An Extracellular Antifungal Chitinase from *Lecanicillium lecanii*: Purification, Properties, and Application in Biocontrol against Plant Pathogenic Fungi. Turk. J. Biol..

[57] Błaszczyk L., Waśkiewicz A., Gromadzka K., Mikołajczak K., Chełkowski J. (2021). Sarocladium and *Lecanicillium* Associated with Maize Seeds and Their Potential to Form Selected Secondary Metabolites. Biomolecules.

[58] Berestetskiy A., Hu Q. (2021). The Chemical Ecology Approach to Reveal Fungal Metabolites for Arthropod Pest Management. Microorganisms.

[59] Shoresh M., Harman G.E., Mastouri F. (2010). Induced Systemic Resistance and Plant Responses to Fungal Biocontrol Agents. Annu. Rev. Phytopathol..

[60] Kholida N., Prihatiningsih N., Mugiastuti E., Nurchasanah S. (2025). Correlation of Various Environmental Factors to Coffee Leaf Rust Disease and Its Natural Control. IRAQI J. Agric. Sci..

[61] Das D.K., Machenahalli S., Giri M.S., Ranjini A.P., Rao N.S., Shivanna M.B. (2024). Efficacy of Biological Agent *Lecanicillium lecanii* for the Management of Coffee Leaf Rust in India. Indian Phytopathol..

[62] Cruz D., Jaramillo-Riofrío A., Herrera P., Aguinsaca R., Chamba M. (2024). Fungal Diversity Detected by ITS-5.8S from *Coffea arabica* Leaves Infected by Rust (*Hemileia vastatrix*) in Southern Ecuador. Diversity.

[63] Colmán A.A., Araújo J.P.M., Evans H.C., Mansur P.S.C., Salcedo-Sarmiento S., Silva A.L., Kapeua-Ndacnou M., Belachew-Bekele B.K., Pereira C.M., Crous P.W. (2025). Hidden Diversity behind the *Lecanicillium*—Like White Colony-Forming Mycoparasites on *Hemileia vastatrix* (Coffee Leaf Rust). Persoonia-Mol. Phylogeny Evol. Fungi.

[64] Vandermeer J., Perfecto I., Liere H. (2009). Evidence for Hyperparasitism of Coffee Rust (*Hemileia vastatrix*) by the Entomogenous Fungus, *Lecanicillium lecanii*, through a Complex Ecological Web. Plant Pathol..

[65] Park M.-J., Hong S.-B., Shin H.-D. (2016). *Lecanicillium uredinophilum* sp. Nov. Associated with Rust Fungi from Korea. Mycotaxon.

[66] Setiawati R., Widiastuti A., Wibowo A., Priyatmojo A. (2021). Variability of *Lecanicillium* Spp. Mycoparasite of Coffee Leaf Rust Pathogen (*Hemileia vastatrix*) in Indonesia. Pak. J. Biol. Sci. PJBS.

[67] Berlanga-Padilla A.M., Lino-López G.J., Ayala-Zermeño M.A., Muñiz-Paredes F., Montesinos-Matías R., Sánchez-González J.A. (2025). Fungi Associated with Coffee Rust *Hemileia vastatrix* and the Description of Two New Species. Fungal Biol..

[68] Nicoletti R., Becchimanzi A. (2020). Endophytism of *Lecanicillium* and *Akanthomyces*. Agriculture.

[69] Deping W. (2018). *Lecanicillium uredinophilum* Known from Rusts, Also Occurs on Animal Hosts with Chitinous Bodies. Asian J. Mycol..

[70] Meng Y., Wellabada Hewage Don P.I.D., Wang D. (2022). A New Strain of *Lecanicillium uredinophilum* Isolated from Tibetan Plateau and Its Insecticidal Activity. Microorganisms.

[71] Arnold A.E. (2007). Understanding the Diversity of Foliar Endophytic Fungi: Progress, Challenges, and Frontiers. Fungal Biol. Rev..

[72] de Bary A. (1866). Morphologie und Physiologie der Pilze, Flechten und Myxomyceten.

[73] Martínez-de-Jesús J. (2025). Strains of *Akanthomyces uredinophilum*, *Simplicillium lanosoniveum*, and *Trichoderma* Spp. Exhibit High Endophytic Activity and Induce Improved Growth of Coffee Plants. Sci. Agropecu..

[74] de Resende M.L.V., Pozza E.A., Reichel T., Botelho D.M.S. (2021). Strategies for Coffee Leaf Rust Management in Organic Crop Systems. Agronomy.

[75] Haddad F., Maffia L.A., Mizubuti E.S.G., Teixeira H. (2009). Biological Control of Coffee Rust by Antagonistic Bacteria under Field Conditions in Brazil. Biol. Control.

[76] Harman G.E., Howell C.R., Viterbo A., Chet I., Lorito M. (2004). *Trichoderma* Species—Opportunistic, Avirulent Plant Symbionts. Nat. Rev. Microbiol..

[77] Del Carmen H Rodríguez M., Evans H.C., de Abreu L.M., de Macedo D.M., Ndacnou M.K., Bekele K.B., Barreto R.W. (2021). New Species and Records of *Trichoderma* Isolated as Mycoparasites and Endophytes from Cultivated and Wild Coffee in Africa. Sci. Rep..

[78] Mamani-Huayhua G., Leon-Ttacca B., Palao-Iturregui L.A., Borja-Loza Y.R., Mamani-Huayhua G., Leon-Ttacca B., Palao-Iturregui L.A., Borja-Loza Y.R. (2021). Biocontrol de La Roya Amarilla Del Cafeto (*Hemileia vastatrix* Berk. & Br.) Con Cepas de *Trichoderma* Sp. Endófito. Cultiv. Trop..

[79] Enríquez-López S.L., Alvarado-Castillo G., Cerdán-Cabrera C.R., Argumedo-Delira R., Escamilla-Prado E. (2025). Control de la roya del café (*Hemileia vastatrix*) con *Trichoderma* spp.: Evaluación cuantitativa mediante el paquete Pliman. Rev. Mex. Fitopatol..

[80] Salcedo-Sarmiento S., Aucique-Pérez C.E., Silveira P.R., Colmán A.A., Silva A.L., Corrêa Mansur P.S., Rodrigues F.Á., Evans H.C., Barreto R.W. (2021). Elucidating the Interactions between the Rust *Hemileia vastatrix* and a Calonectria Mycoparasite and the Coffee Plant. iScience.

[81] Jackson D., Skillman J., Vandermeer J. (2012). Indirect Biological Control of the Coffee Leaf Rust, *Hemileia vastatrix*, by the Entomogenous Fungus *Lecanicillium lecanii* in a Complex Coffee Agroecosystem. Biol. Control.

[82] Subramani D., Rajanaika R. (2009). Biological Control of Coffee Leaf Rust Pathogen, *Hemileia vastatrix* Berkeley and Broome, Using *Bacillus subtiles* and *Pseudomonas fluorescens*. J. Biopestic..

[83] Fajardo-Franco M.L., Aguilar-Tlatelpa M., Guzmán-Plazola R.A., Fajardo-Franco M.L., Aguilar-Tlatelpa M., Guzmán-Plazola R.A. (2020). Biofungicides Evaluation in Two Coffee Cultivars for *Hemileia vastatrix* Control. Rev. Mex. Fitopatol..

[84] Alves J.R., Celestino F.N., Moraes A.G.d., Reis R.A.d., Grecco E.D. (2023). Growth Promoting Fungi Increase the Quality of *Coffea canephora* Seedlings Pierre Ex a. Froehner. Coffee Sci..

[85] Abbaszadeh G., Dhillon M., Srivastava C., Gautam R. (2011). Effect of Climatic Factors on Bioefficacy of Biopesticides in Insect Pest Management. Biopestic. Int..

[86] Nguyen H.-T., Pham T.-T., Nguyen P.-T., Dinh N.-C.-G., Le M.-T., Nguyen T.-D., Nguyen T.-T., Nguyen V.-B. (2025). Microbial Biocontrol in Agriculture: From Mechanistic Understanding to Field Application. Discov. Plants.

[87] Wei Z., Huang J., Yang T., Jousset A., Xu Y., Shen Q., Friman V.-P. (2017). Seasonal Variation in the Biocontrol Efficiency of Bacterial Wilt Is Driven by Temperature-Mediated Changes in Bacterial Competitive Interactions. J. Appl. Ecol..

[88] Koutouleas A., Collinge D.B., Ræbild A. (2023). Alternative Plant Protection Strategies for Tomorrow’s Coffee. Plant Pathol..

[89] Fenice M. (2016). The Psychrotolerant Antarctic Fungus *Lecanicillium muscarium* CCFEE 5003: A Powerful Producer of Cold-Tolerant Chitinolytic Enzymes. Molecules.

[90] Manfrino R., Gutierrez A., Diez del Valle F., Schuster C., Ben Gharsa H., López Lastra C., Leclerque A. (2022). First Description of *Akanthomyces uredinophilus* Comb. Nov. from Hemipteran Insects in America. Diversity.

[91] Silva M.d.C., Guerra-Guimarães L., Diniz I., Loureiro A., Azinheira H., Pereira A.P., Tavares S., Batista D., Várzea V. (2022). An Overview of the Mechanisms Involved in Coffee-*Hemileia vastatrix* Interactions: Plant and Pathogen Perspectives. Agronomy.

[92] Ongena M., Jacques P. (2008). *Bacillus* Lipopeptides: Versatile Weapons for Plant Disease Biocontrol. Trends Microbiol..

[93] Teixidó N., Usall J., Torres R. (2022). Insight into a Successful Development of Biocontrol Agents: Production, Formulation, Packaging, and Shelf Life as Key Aspects. Horticulturae.

[94] Askary H., Benhamou N., Brodeur J. (1999). Ultrastructural and Cytochemical Characterization of Aphid Invasion by the Hyphomycete *Verticillium lecanii*. J. Invertebr. Pathol..

[95] Chen C., Li M., Gao R., Yan M., Zhou T., Tang Y., Li J. (2026). Functional Study of the Chitinase CaChi93 Gene from the Mycoparasitic *Cladosporium* sp. SYC23. J. Fungi.

[96] Adnan M., Islam W., Shabbir A., Khan K.A., Ghramh H.A., Huang Z., Chen H.Y.H., Lu G. (2019). Plant Defense against Fungal Pathogens by Antagonistic Fungi with *Trichoderma* in Focus. Microb. Pathog..

[97] Rabbee M.F., Ali M.S., Islam M.N., Rahman M.M., Hasan M.M., Baek K.H. (2024). Endophyte Mediated Biocontrol Mechanisms of Phytopathogens in Agriculture. Res. Microbiol..

[98] Ayalew B., Hylander K., Adugna G., Zewdie B., Tack A.J.M. (2024). Impact of Climate on a Host–Hyperparasite Interaction on Arabica Coffee in Its Native Range. J. Appl. Ecol..

[99] Perazzolli M. (2015). Impact of the Omic Technologies for Understanding the Modes of Action of Biological Control Agents against Plant Pathogens. BioControl.

[100] Velivelli S.L.S., Vos P.D., Kromann P., Declerck S., Prestwich B.D. (2014). Biological Control Agents: From Field to Market, Problems, and Challenges. Trends Biotechnol..

